# The impact of periodontal disease on the clinical outcomes of COVID-19: A systematic review and meta-analysis

**DOI:** 10.1186/s12903-023-03378-0

**Published:** 2023-09-09

**Authors:** Sadeq Ali Al-Maweri, Mohammed Nasser Alhajj, Esam Halboub, Faleh Tamimi, Nosizana Mohd Salleh, Mohammed Sultan Al-Ak’hali, Saba Kassim, Saleem Abdulrab, Lamyia Anweigi, Marwan Mansoor Ali Mohammed

**Affiliations:** 1 College of Dental Medicine, QU Health, Qatar University, Doha, Qatar; 2 Department of Restorative Dentistry, Faculty of Dentistry, University of Malaya, Federal Territory of Kuala Lumpur, Kuala Lumpur, Malaysia; 3 Department of Prosthodontics, Faculty of Dentistry, Thamar University, Dhamar, Yemen; 4 Department of Maxillofacial Surgery and Diagnostic Sciences, College of Dentistry, Jazan University, Jazan, Saudi Arabia; 5 Department of Preventive Dental Sciences, College of Dentistry, Jazan University, Jazan, Saudi Arabia; 6 Department of Preventive Dental Sciences, College of Dentistry, Taibah University, Al-Madinah Al-Munawwarah, Saudi Arabia; 7 Al Khor Health Center, Primary Health Care Corporation, Doha, Qatar; 8 Department of Oral and Craniofacial Health Sciences, College of Dental Medicine, University of Sharjah, Sharjah, United Arab Emirates

**Keywords:** COVID-19 outcomes, Periodontal disease, Association, Systematic review

## Abstract

**Background:**

A possible relationship between periodontitis (PD) and COVID-19 and its adverse outcomes has been suggested. Hence, the present systematic review and meta-analysis aimed to investigate the available evidence regarding the potential association between periodontitis (PD) and COVID-19 and its adverse outcomes.

**Materials and methods:**

PubMed, Scopus, Web of Science, and Google Scholar were searched for relevant studies published up to April 15^th^, 2023. Studies that evaluated the association between PD and COVID-19 were included. Risk of bias was evaluated by two reviewers, and meta-analyses were performed using RevMan 5.3 software.

**Results:**

A total of 22 studies involving 92,535 patients from USA, Europe, Asia, the Middle East and South America were included; of these, 12 were pooled into the meta-analysis. Most of the studies (19 studies) reported a significant association between PD and COVID-19. The pooled data found a significant association between PD and COVID-19 outcomes: more severe symptoms (OR = 6.95, *P* = 0.0008), ICU admissions (OR = 3.15, *P* = 0.0001), and mortality (OR = 1.92, *P* = 0.21). Additionally, compared to mild PD, severe PD was significantly associated with higher risks of severe COVID-19 outcomes: severe symptoms (*P* = 0.02); ICU admission (*P* = 0.0001); and higher mortality rates (*P* = 0.0001). The results also revealed 58% higher risk for COVID-19 infection in patients with PD (*P* = 0.00001).

**Conclusions:**

The present findings suggest a possible association between poor periodontal health and the risk of poor COVID-19 outcomes. However, owing to the observed methodological heterogeneity across the included studies, further prospective cohort studies with standardized methodologies are warranted to further unravel the potential association between periodontal disease and COVID-19 and its adverse outcomes.

**Supplementary Information:**

The online version contains supplementary material available at 10.1186/s12903-023-03378-0.

## Background

The Corona virus disease of 2019 (COVID-19) caused by the SARS-CoV-2 virus has resulted in an enormous impact on the global health and economy [1–3]. Despite the fact that most COVID-19 patients recover without major complications, few patients still suffer severe complications including multiple organ failure and death [4, 5]. Such complications are driven by serious conditions triggered by the infection such severe acute respiratory distress, systemic inflammatory reactions, and coagulopathies [6, 7]. It became obvious that several comorbidities such as obesity, hypertension, diabetes and advanced age are associated with severe COVID-19 [4–6, 8]. A considerable fraction of apparently healthy and young patients, with no clear identifiable risk factors, however, still develops severe complications.

Periodontitis (PD), the most common form of periodontal diseases, is a chronic disease involving the inflammation and subsequent damage of the tissues surrounding the teeth [9]. If not treated properly, PD leads to the destruction of the bone surrounding the teeth and ultimately loss of the teeth themselves [10]. Beyond such local consequences, PD can also have detrimental effects on systemic health [10]. Growing evidence links PD to several systemic diseases including diabetes mellitus, cardiovascular diseases, pneumonia, metabolic syndrome, and cancers [9, 11–17]. Such systemic effects of PD might be ascribed to the bacterial products and inflammatory mediators in the periodontal infected tissues that can reach the blood stream and increase the systemic inflammatory burden [18]. Worth to mention that periodontal pathogens can reach the respiratory tract, aggravating, and/or even initiating respiratory infections [17].

Given that PD shares with COVID-19 many features including comorbidities and increased blood levels of inflammation and coagulation biomarkers, [19, 20] several researchers have hypothesized that PD could be associated with a higher risk of COVID-19, and the development of its adverse outcomes [20, 21]. Marouf et al. reported a higher risk of COVID-19 complications including death (OR = 8.81), intensive care unit (ICU) admission (OR = 3.54), and the need for assisted ventilation (OR = 4.57) in patients with periodontitis [22]. Gupta et al. reported that the likelihood of requiring assisted ventilation, hospital admission, death, and getting COVID-19-related pneumonia were 7.45, 36.52, 14.58, and 4.42 folds, respectively, in COVID-19 patients with PD compared to COVID-19 patients without PD [23]. Additionally, 2022 case–control study among US COVID-19 patients reported significantly greater missing teeth and alveolar loss tooth in Covid-19 positive patients and in those with severe complications [24]. Interestingly, a 2022 case–control study by Said et al. reported a significant association between history of periodontal therapy and favorable COVID-19 outcomes, indicating a positive role of periodontal therapy on Covid-19 complications [25]. Using a two-sample Mendelian randomization, Wang et al. revealed that PD was significantly associated with higher risk for COVID-19 infection and severe complications [26]. Conversely, two studies with a large sample size from UK Biobank cohorts did not support any significant association between PD and the risk and outcomes of COVID-19 [27, 28]. Similarly, a retrospective cross-sectional Dutch study did not find a significant association between alveolar bone loss and complications of COVID-19 [29]. One systematic review of two studies reported a significant association between PD and COVID-19 [30]. A more recent systematic review and meta-analysis by Baima et al., [31] investigating the potential link between PD and Covid-19, reported a significant association between PD and Covid-19 susceptibility and poor outcomes [31]. Nevertheless, the latter review included only very limited studies (n = 8; studies published up till Feb. 2022), and since then numerous relevant studies were published with interesting findings. Additionally, the latter two systematic reviews did not in-depth assess the correlation between severity of PD and severity of Covid-19 symptoms. Therefore, this systematic review and meta-analysis sought to comprehensively analyze and summarize the available evidence on the association between PD and COVID-19. The focused question was: Does periodontal health status have an impact on COVID-19 risk and clinical outcomes?

## Materials and methods

The present systematic review and meta-analysis adhered strictly to the PRISMA 2020 guidelines and followed utterly PECO principles. This systematic review was registered in Open Science Framework (OSF) registries (https://doi.org/10.17605/OSF.IO/KW7TC). The PECO research question was: Is periodontal disease a risk factor for COVID-19 and its poor outcomes?

### Eligibility criteria

All studies (cohort, case–control, and cross-sectional and interventional studies) that assessed the association of periodontal diseases with COVID-19 outcomes in humans were eligible.

*Exposure*: periodontal disease parameters.

*Outcome*: COVID-19 infection and/or its associated adverse outcomes.

Case reports, post-mortem studies, animal and experimental studies, review articles, commentaries, and studies with unclear exposures/outcomes were excluded.

### Search strategy and information sources

An extensive search of online databases (PubMed, Scopus, and Web of Science) and Google Scholar search engine, supplemented with manual search was conducted independently by two reviewers (SA and MA) on April 16^th^, 2023 for all relevant studies published from December 2019 up to April 15^th^, 2023. The following Mesh terms and free keywords were used: ("Periodontal Diseases"[Mesh] OR "Oral Health"[Mesh] OR “Periodontal disease” OR periodontitis” OR Periodont* OR periodontal pathogen*) AND ("SARS-CoV-2″[Mesh] OR COVID-19). Detailed information about the search strategies is presented in Supplementary Table 1.

### Screening and selection process

All retrieved studies were exported to the EndNote program, which eased removal of duplicates. Then, the titles and abstracts of all articles were screened independently by two reviewers (SA and FT), and irrelevant articles were excluded. The full-texts of all potentially eligible articles were independently evaluated by the two reviewers for inclusions, and irrelevant articles were eliminated.

### Data extraction

All relevant data were independently extracted by two investigators (SA, MA) using customized forms. The extracted data included the following: first author name, country of the study, study design, sample size, age and gender of the participants, periodontal variables (exposure) and COVID-19 variables (the main outcomes).

### Quality assessment

Two reviewers (SA and MA) assessed independently the quality of all included studies using the Newcastle Ottawa Scale (NOS) for assessing the quality of non-randomized studies [32]. Disagreements, if any, were resolved by discussion**.** The overall quality of each study was rated as either: high quality, 7–9 stars; moderate quality, 4–6 stars; or poor quality, 0–3 stars [32].

### Statistical analysis

The Review Manager (RevMan) Version 5.3 software was used for data analysis. The pooled odds ratios (ORs) along with 95% confidence intervals (CIs) were used to calculate the risk of COVID-19 and associated outcomes in patients with PD and periodontium healthy patients. Heterogeneity was evaluated using the Chi-square test and the I^2^ statistics. A Fixed-effects model was used for low/moderate heterogeneity (I^2^ ≤ 50%), while a random effect model was applied for significant heterogeneity (I^2^ > 50%).

Se nsitivity tests: due to the limited number of analyzed studies, no sensitivity tests were done.

## Results

### Study selection

**Fig. 1:**
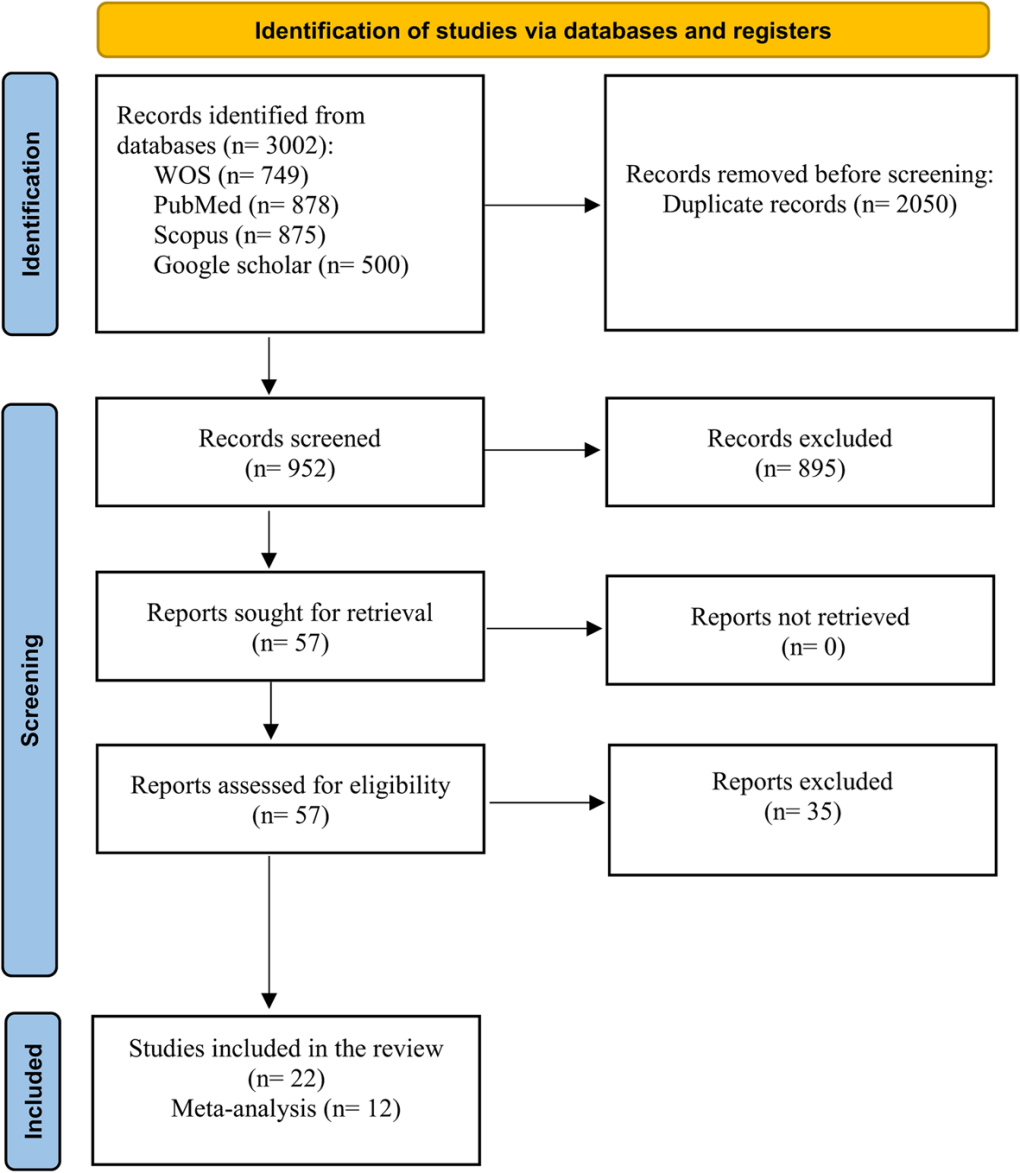
Flowchart of the search strategy

Figure 1 presents the search strategy of the present study. The initial online searches yielded 3002 articles, of which 2050 duplicate records were excluded. The titles and abstracts of 952 articles were screened for eligibility, and 895 were found irrelevant and thus excluded. The full-texts of the remaining 57 articles were assessed for eligibility, and 35 were excluded for various reasons (Supplementary Table 2). Eventually, 22 studies were included in the present systematic review, and 12 were pooled into the meta-analysis.

### General characteristics of the included studies

A total of 22 studies, involving 92,535 patients were included in the present systematic review; [22–25, 27–29, 33–47] of these, 12 studies were included in the meta-analysis [23, 25, 27, 28, 33, 35, 36, 40, 41, 43, 44, 46, 47]. Twelve of these studies were case–control studies, [22–25, 29, 34, 35, 37, 38, 42, 44, 46] three cohort studies, [27, 28, 39] and seven cross-sectional studies [33, 36, 40, 41, 43, 45, 47]. Six studies were conducted in India [23, 34, 38, 40, 42, 44] three in the UK [27, 28, 39], two in Brazil [35, 36] two in Saudi Arabia [33, 43], two in Qatar [22, 25], two in Turkey [46, 47] and one each in: the Netherlands, [29]. Spain, [45] Egypt, [41] Mexico, [37] and the USA [24]. The mean age of the participants ranged from 38.2 to 68.6 years, with almost equal representation of both genders. Diagnosis of COVID-19 was confirmed by PCR test in all of the included studies.

### Periodontal parameters (Exposure)

Ascertainment of periodontal parameters were highly variable across the included studies. One or more of the following periodontal parameters were considered: number of missing teeth, pocket depth, gum bleeding, alveolar bone loss, and loose teeth (Table 1). Similarly, ascertainment of the exposure methods varied greatly across the included studies: clinical examination in nine studies, [23, 34–36, 38, 40, 42, 44, 46] self-reported in five studies, [27, 28, 37, 39, 41] and dental radiographs in eight studies [22, 24, 25, 29, 33, 43, 45, 47].

**Table 1:** General characteristics of the included studies

**Author (country)**	**Study** **Design**	**No of Participants M/F (age)**	**Perio-related parameters assessment (Exposure)**	**COVID-19 Outcome parameters (Outcomes)**	**confounding factors**
Anand et al., 2021 [22] (India)	Case-control	Total *N*= 150; Gender: 85M/65 F Cases: COVID-19 positive patients (*n*=79) (Mean age: 43.34) Controls: COVID-19 negative (*n*=71) (Mean age: 38.24)	PI, CI, PD, GB, tooth mobility, recession, CAL (clinical exam)	COVID-19 infection risk	Tobacco use, age, systemic diseases, oral hygiene practices Diabetes Hypertension Neoplasma
Marouf et al., 2021 [25] (Qatar)	Case-control	Total *N*= 568; Gender: 310/258 Cases: COVID-19 patients with complications (*n*=40) (mean age: 53.5 ys) Control: COVID-19 patients without complications (*n*=528) (mean age: 41.5)	Interdental bone loss Periodontal condition (radiographs)	Risk of COVID-19 infection, COVID-19 complications: ICU, death, assisted ventilation	age, sex, smoking, BMI, diabetes and co-morbidities.
Larvin et al., 2020 [29] (UK biobank)	Retrospective cohort	Total N: 13,253 biobank (1,616 positive covid and 11,637 negative ) Gender: 6451/6,802 Age: 68.55 years Cases: patients with PD (*n*= 2100) Controls: patients with no PD (*n*= 11,153)	Painful/bleeding gums Loose teeth (Self-reported)	Risk of COVID-19, COVID-19 complications -Hospital admission - mortality	age, sex, ethnicity, income, smoking, BMI, cancer, diabetes and CVD
Gupta et al., 2021 [23] (India)	Case-control	Total *n*= 82; gender: 48/34 Cases: 55 COVID-19 patients with PD (*n*= 55) Controls: 27 COVID-19 patients with no PD (*n*=27) Mean Ages: 34.44 to 62.94	Pocket depth, CAL, BOP Gingivitis, Periodontitis( stage I, stage II, stage III, stage IV) and Recession (Clinical exam)	pneumonia, death/survival, - hospital admission-assisted ventilation	age, sex, smoking habits , diabetes, hypertension, pulmonary disease, chronic kidney disease, cancer, CAD, obesity
Holt et al., 2021 [40] (UK)	Cohort study	15227 participants (446 COVID-19 cases) Gender: 4597/10630 Age: 59.4 years	Painful/bleeding gums Loose teeth (Self-reported)	Risk of COVID-19 infection	age, sex, alcohol and smoking habits, ethnicity, co-morbidities BMI
Donders et al., 2022 [31] (Netherlands)	Case-control study	Total : *N*=133 Gender: 61/72 Age: 61.7 years G1: Mild COVID-19 (*n*=82) G2: moderate (*n*=30) G3: Severe COVID-19 (*n*=21)	-Periodontitis -tooth loss (Radiographs)	Severity of COVID = -Mild, -moderate and, Severe (ICU/death, ICU)	age, sex, smoking, diabetes, CVD, kidney diseases
Larvin et al., 2021 [30] (UK)	Retrospective cohort study periodontal disease in obesity on COVID-19	Total *n*= 58,897; 14,466 (24.6%) COVID-19 positive PD patients: *n*= 9332 No PD: *n*= 49,565 age; 56.83 y 53% females	bleeding gums, painful gums, and loose teeth (Self-reported)	risk of COVID-19 infection, hospital admission, and mortality	age, sex, ethnicity, income, smoking, BMI, cancer, diabetes and CVD
Costa et al., 2022 [37] (Brazil)	Prospective Cross- sectional study	Total *N*=128 COVID-19 positive Gender 68/60 Age: 58.7y	DMFT) index, Probing depth (PD), clinical attachment level (CAL), bleeding on probing (Clinically)	-Severity (mild, moderate, severe, or critical) -Hospital admission -discharge or death -ventilation	Patient age Comorbidities((diabetes, hypertension, and obesity)
Kaur et al., 2022 [42] (India)	Case-control	N=116 subjects suffering from COVID-19 Gender 70/46 Two groups G1: Moderate COVID-19 cases (G2: Mild COVID-19	Periodontitis(Stage 0-1) Periodontitis(Stage 2-3) (Clinically)	Severity of COVID = -Mild, -moderate	Smoking, diabetes, age, comorbidities, BMI
Mishra et al., 2022 [44] (India)	Case-control	N= 294 subject with Covid Gender 154/140 COVID-19 cases were divided into: G1: Mild cases (163) Moderate cases (83) Severe cases (48)	Periodontitis (Stage 1-1I) Periodontitis (Stage III-IV) PI, CI, PD, GB, tooth mobility, recession, CAL (Clinically)	Severity of COVID = -Mild, -moderate, severe	Smoking, diabetes, age, gender, BMI, oral hygiene practices
Kamel et al., 2021 [24] (Egypt)	Cross-sectional	*N*= 308 Age: 18-55 years No gender reported	Gum bleeding, tooth loss, gum pain (Self-reported)	Severity of COVID-19 (sever and mild) (dyspenia, low O2 saturation, hospitalization),	Participants with known risk factors and comorbidities that could affect COVID-19 severity were excluded.
Sirin & Ozcelik, 2021 [47] (Turkey)	Comparative Cross-sectional	*N*= 137 Age: 20-65 yrs Gender: 71/66	Alveolar bone loss, tooth loss, Dental implants Apical periodontitis (Radiographs)	Severity of COVID-19	NA
Koppolu et al., 2023 [43] (KSA)	Retrospective comparative Cross-sectional	*N*= 196 Age: 18-60 yrs Gender: 62/36	Alveolar bone loss (Radiographs)	Severity of COVID-19 (COVID negative, Home isolation, Hospital without ventilator and ICU, Ventilation, ICU, Death)	Age, sex, smoking, comorbidity.
Sari et al., 2023 [46] (Turkey)	Case-control	*N*= 80 (50% had COVID) Age: 23-64 years Gender: 38/42	PD, CAL, PI, GI, BOP (Clinically)	Prevalence of COVID-19 infection among patients with periodontitis.	Age, gender, smoking, BMI.
Said et al., 2023 [27] (Qatar)	Case-control	*N*= 1325 (71 suffered severe COVID-19 complications resulting in ICU admission, mechanical ventilation and/or death.) Age: > 18 years Gender: 577/748	Inter-dental bone loss in posterior teeth (Radiographs)	COVID-19 complications (any complication, ICU admission, ventilation and death)	Age, sex, diabetes, smoking habits, BMI and comorbidities
Alnomay et al., 2023 [35] (KSA)	Retrospective cross-sectional	*N*= 188 Age: ≥ 18 years Gender: 81/107	Inter-dental bone loss in posterior teeth (Radiographs)	Severity of COVID-19	Age, gender, smoking, diabetes, hypertension, obesity, and comorbidities
Guardado-Luevanos et al., 2022 [38] (Mexico)	Case-control	N= 234 Age: 20-75 years Gender: 96/138	periodontal disease (Self-reported)	Prevalence of COVID-19 infection among patients with self-reported periodontitis.	Age, sex, smoking, and comorbidities.
Gujar et al., 2022 [39] (India)	Case-control	*N*= 150 (79 were categorized as cases) Age: ≥ 18 years No gender reported	Plaque scores, calculus scores, tooth mobility, gingival bleeding, probing depth (PD), recession (REC), and clinical attachment level (CAL) (Clinically)	Prevalence of COVID-19 infection among patients with periodontitis.	Age, sex, smoking, oral hygiene habits, and comorbidities.
Wadhwa et al., 2022 [26] (USA)	Case-control	*N*= 387 COVID-19 cases, 387 control Age: 47.1 years Gender: 260/514	Missing teeth Alveolar bone (Radiographs)	-Risk of Covid-19 -Severity of Covid-19	Age, gender, smoking, race, ethnicity
Poyato-Borrego et al., 2023 [45] (Spain)	Retrospective comparative Cross-sectional	*N*= 52 Age: 52.3 Gender: 30/22	Alveolar bone loss (Radiographs)	Severity of COVID-19; mild/moderate disease (MMG) and severe/critical disease (SCG)	
Kalsi et al., 2023 [41] (India)	Cross-sectional	*N*= 44 Age: 20-50 years Gender: 23/21	Probing pocket depth (PPD), clinical attachment level (CAL), bleeding on probing (BOP), periodontal inflamed surface area (PISA) (Clinically)	Severity of COVID-19	NA
Bemquerer et al., 2023 [36] (Brazil)	Case-control	*N*= 99 cases; 182 control Age: 16-74 yrs cases; 20-74 yrs control Gender: 52/47 cases; 96/86 control	Plaque index, probing depth (PD), clinical attachment level (CAL), and bleeding on probing (BOP). (Clinically)	Severity of COVID-19 (use of oxygen, hospitalization, admission to the intensive care unit (ICU), admission to the semi-intensive care unit (SICU), use of mechanical ventilation, length of hospitalization, length of ICU admission, length of mechanical ventilation, and death)	Age, sex, schooling, family income, toothbrushing, flossing, smoking, and BMI.

*M* male, *F* female, *PD* periodontal disease, *CAD* coronary artery disease, *CVD* Cardiovascular disease, *NM* not available

### Outcome measures

Most of the studies reported on adverse outcomes of Covid-19. Ascertainment of the adverse outcomes of COVID-19 included one or more of the following: severity of symptoms, ICU admission, hospital admission, and mortality (Table 1). Nine studies [24, 26–28, 34, 37–39, 46] also reported on the risk of covid-19 in periodontitis patients.

### Qualitative results

The majority of the studies (19 studies) found a significant association between PD and COVID-19 adverse outcomes (i.e., severity of symptoms, hospital admission, ICU and mortality). Conversely, three studies did not report a significant association between PD and COVID-19 adverse outcomes [27–29] except for mortality in one study [28]. Concerning susceptibility to COVID-19, nine studies [26–28, 34, 37–39, 46] reported on this outcome; six of these studies reported a significant association between having PD and the risk for COVID-19 infection, [24, 26, 34, 37, 38, 46] whereas the other three studies [27, 28, 39] didn’t confirm such results (Table 2).

**Table 2:** Summary of the main outcomes

Study	**Main outcome **
Anand et al., 2021 [22]	There was a highly significant association between some PD parameters and the risk of COVID-19 : plaque index (OR 7.01; 95% CI, 1.83 -26.94), gingival inflammation (OR, 17.65; 95% CI, 5.95 - 52.37), CAL (OR, 8.46; 95% CI, 3.47 to 20.63), periodontitis (OR, 11.75; 95% CI, 3.89 to 35.49).yet, there were no significant differences between the two groups in terms of missing teeth, carious teeth, and calculus scores.
Marouf et al., 2021 [25]	A significant association between periodontitis and COVID-19 complications including death (OR = 8.81, 95% CI 1.00–77.7), ICU admission (OR = 3.54, 95% CI 1.39–9.05) and the need for assisted ventilation (OR = 4.57, 95% CI 1.19–17.4). Furthermore, white blood cells, D-dimer and CRP levels were significantly higher in COVID-19 patients with PD.
Larvin et al 2020 [29]	There was no any association between painful/bleeding gum and the risk of COVID-19 (OR = 1.10, 95% CI = 0.72–1.69) or risk of hospital admission (OR = 0.90, 95% CI = 0.59–1.37). However, there was a significant association between PD and the mortality rate; participants with painful/bleeding gum had twofold higher mortality rate than those with healthy gum (OR = 1.71, 95% CI = 1.05–2.72). There was no a significant association between loose teeth with COVID-19 parameters: risk of COVID-19 (OR = 1.15, 95% CI = 0.84–1.59); hospital admission (OR = 1.55, 95% CI = 0.87–2.77); or mortality (OR = 1.85; 95% CI = 0.92–2.72)
Gupta et al. 2021 [23]	There is a significant association between PD and COVID-19 - outcomes. Higher severity of PD was associated with 7.45 odds of requiring assisted ventilation, 36.52 odds of hospital admission, 14.58 odds of death, and 4.42 odds of COVID-19 -related pneumonia
Holt et al. 2021 [40]	The results showed a significant increase in the risk of COVID-19 in PD subjects (OR: 1.20). However, after controlling all potential confounding factors, no significant association was observed between the two conditions.
Donders et al 2022 [31]	The results showed a significant association between PD (alveolar bone loss [OR: 5.60; 95%CI: 1.21; 25.99; *P* = 0.028] and tooth loss [OR: 1.04; 95%CI: 1.00; 1.09; *P* = 0.047]) with severity of COVID-19. However, such associations disappeared after adjusting for all potential confounders (P more than 0.05).
Larvin et al 2021 [30]	The risk for COVID-19 infection was not associated with periodontal disease across the 3 BMI categories: normal weight (OR, 0.97; 95% CI, 0.88 to 1.07), overweight (OR, 1.06; 95% CI, 0.98 to 1.15), and obese (OR, 1.08; 95% CI, 0.99 to 1.17). The risk of hospital admission for people with periodontal disease was 38% higher in participants who were overweight (HR, 1.38;95% CI, 1.02 to 1.87) and 124% higher in those who were obese (HR, 2.24; 95% CI, 1.66 to 3.03) compared to those of normal weigh. In addition, for participants with obesity, the mortality rate was much higher (hazard ratio, 3.11; 95% CI, 1.91 to 5.06) in participants with periodontal disease than those with healthy periodontium.
Costa et al., 2022 [37]	Periodontitis was significantly associated with ICU admission [IRR = 1.44 (95%CI = 1.07–1.95); *p* = 0.017], critical symptoms [IRR = 2.56 (95%CI = 1.44–4.55); *p* = 0.001], and risk of death [IRR = 2.05 (95%CI = 1.12–3.76); *p* = 0.020]
Kaur et al 2022 [42]	The risk of severe periodontal disease was 6.32 times higher in COVID-19 patients with severe symptoms (*P*= 0.024) as compared to mild COVID cases.
Mishra et al 2022 [44]	There was a significant association between periodontitis and severity of COVID-19 symptoms. The adjusted odds ratio (OR) of having severe COVID-19 in periodontitis patients was 2.8133 (0.4077–19.7523 at 95% CI, *p* = 0.004).
Kamel et al. 2021 [24]	There was a significant association between poor oral health and the severity of COVID-19. Additionally, poor oral health was significantly associated with delayed recovery time and higher CRP values.
Sirin & Ozcelik 2021 [47]	There was a significant association between dental disease including alveolar bone loss and severity of COVID-19 complications. Individuals with severe dental diseases (including alveolar bone loss) had more severe COVID-19 symptoms and higher mortality rate than those with no/mild dental diseases.
Koppolu et al., 2023 [43]	There was a statistically significant link between gingivitis and periodontitis. The majority of mild gingivitis cases (63%) was associated with the COVID-19-negative group, whereas the majority of severe gingivitis groups (85.7%) was associated with the COVID-19-positive group (χ2 = 9.94; *P* = 0.007). Similarly, the majority of Stage 1 periodontitis (62.9%) was associated with COVID-19-negative participants, whereas the majority of Stage 4 periodontitis (*P* = 0.007) was associated with COVID-19-positive groups (χ2 = 22.51; *P* = 0.047).
Sari et al., 2023 [46]	A higher prevalence of periodontitis compared with gingivitis and periodontal health was detected in the test group (*p*=0.003). In line with this, all clinical periodontal parameters related to periodontal disease severity were higher in the test group than in the control group. In particular, GI, BOP (%), PD, CAL, and the number of missing teeth were statistically significantly higher in the test group than in the control group (*p* < 0.05), except PI, which did not reach statistical significance (*p* = 0.052).
Said et al., 2023 [27]	Risk analysis of COVID-19 complications revealed that while periodontitis stage 2–4 (regardless of treatment) was associated with higher risk of complications (i.e. need for mechanical ventilation [AOR = 3.32, 95% CI 1.10–10.08, *p* = 0.034]), subjects with treated periodontitis had a lower risk than the non-treated ones. Adjusted OR analysis comparing treated and non-treated periodontitis (stages 2–4) revealed that treated patients were at lower risk of complications; however, this was not statistically significant.
Alnomay et al., 2023 [35]	Patients with periodontitis were 3-times 182 more likely to have COVID-19 complications than those without periodontitis (*p* = 0.025).
Guardado-Luevanos et al., 2022 [38]	A statistically significant difference (*p* value < 0.001) was observed, showing that positive self-RPD (*n* = 95, 85.1%) was often higher in SARS-CoV-2-positive individuals than in the controls (*n* = 66, 56.4%), with an OR of 3.3 (1.8–6.0) for SARS-CoV-2 infection in people with self-reported periodontal disease.
Gujar et al., 2022 [39]	Participants with COVID-19 had significantly higher mean values of plaque scores, number of mobile teeth, gingival bleeding scores, PD, REC, and CAL compared to the controls. The mean percentages of inter-proximal sites with PD ≥ 4 mm, PD ≥ 5 mm, CAL ≥ 3 mm, CAL ≥ 4 mm, and CAL ≥ 6 mm were also significantly higher in the case group than in the control group.
Wadhwa et al. 2022 [26]	Covid-19 infected patients had significantly greater alveolar bone loss and missing teeth than controls. Additionally, missing teeth and bone loss were associated with more hospitalization.
Poyato-Borrego et al., 2023 [45]	A significant association has been found between the severity of COVID-19 and the periodontal disease (*p*= 0.04).
Kalsi et al., 2023 [41]	The percentage of patients suffering from severe COVID disease was least (8.3%) in stage 1 of periodontitis and was highest (62.5%) in stage 4 of periodontitis. Whereas out of eight patients tested positive in stage 1 of periodontitis, 7 had only mild disease. Out of total of 8 patients with stage 4 category of periodontitis, two remain uninfected, five developed severe form of COVID, and only one patient had moderate COVID disease.
Bemquerer et al., 2023 [36]	In the COVID-19 group, plaque index was worse in patients with periodontitis than in patients without periodontitis (*p* < 0.001). Among individuals with COVID-19, periodontitis was associated with more hospitalization (*p* = 0.009), more days in the ICU (*p* = 0.042), admission to the SICU (*p* = 0.047), and higher need for oxygen therapy (*p* = 0.042). However, there was no difference in total hospitalization length, need for ICU, need for mechanical ventilation, or death between individuals with and without periodontitis in the COVID-19 group. Individuals with periodontitis were 1.13 times more likely to be hospitalized than individuals without periodontitis (CI = 1.01–1.26; *p* = 0.028).

*PD* periodontal disease, *OR* odds ratio, *CI* confidence interval, *MR* Mendelian randomization, *IVW* Inverse-variance weighted, *CRP* C-reactive protein

### Meta-analysis results

#### COVID-19 outcomes in PD versus healthy periodontium patients:

**Fig. 2:**
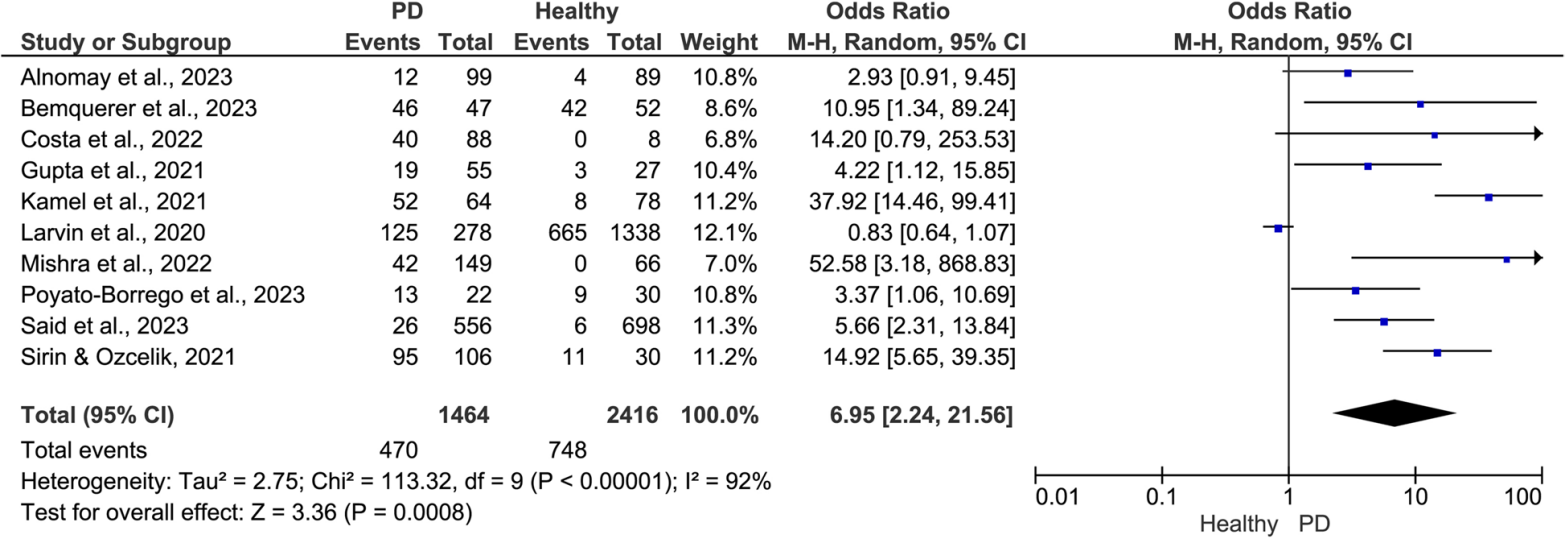
Meta-analysis of the association between periodontal disease (PD) and severe COVID-19 symptoms

**Fig. 3:**
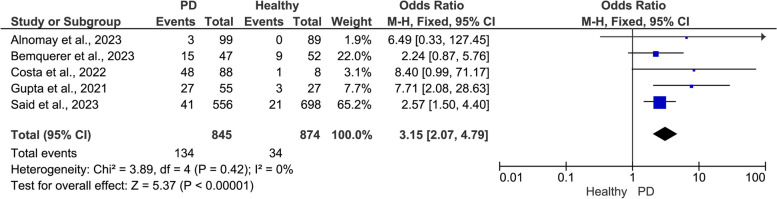
Meta-analysis of the association between periodontal disease (PD) and ICU admissions

**Fig. 4:**
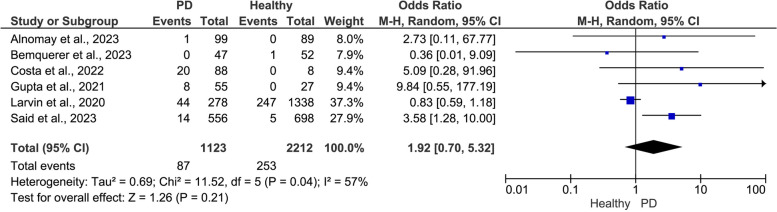
Meta-analysis of the association between periodontal disease (PD) and mortality

The pooled data showed a positive significant association between PD and the risk of adverse COVID-19 outcomes (Figs. 2, 3 and 4). Compared to patients with healthy periodontium, patients with PD showed a significantly higher risk of severe symptoms (OR = 6.95, 95% CI: 2.24, 21.56, I^2^ = 92%, random-effect; *P* = 0.0008)(Fig. 2), ICU admission (OR = 3.15, 95% CI: 2.07, 4.79, I^2^ = 0.00%, fixed-effect; *P* = 0.0001) (Fig. 3), and mortality (OR = 1.92, 95% CI: 0.70, 5.32, I^2^ = 57%, random-effect; *P* = 0.21) (Fig. 4).

#### COVID-19 outcomes by severity of PD (severe PD versus mild PD)

**Fig. 5:**
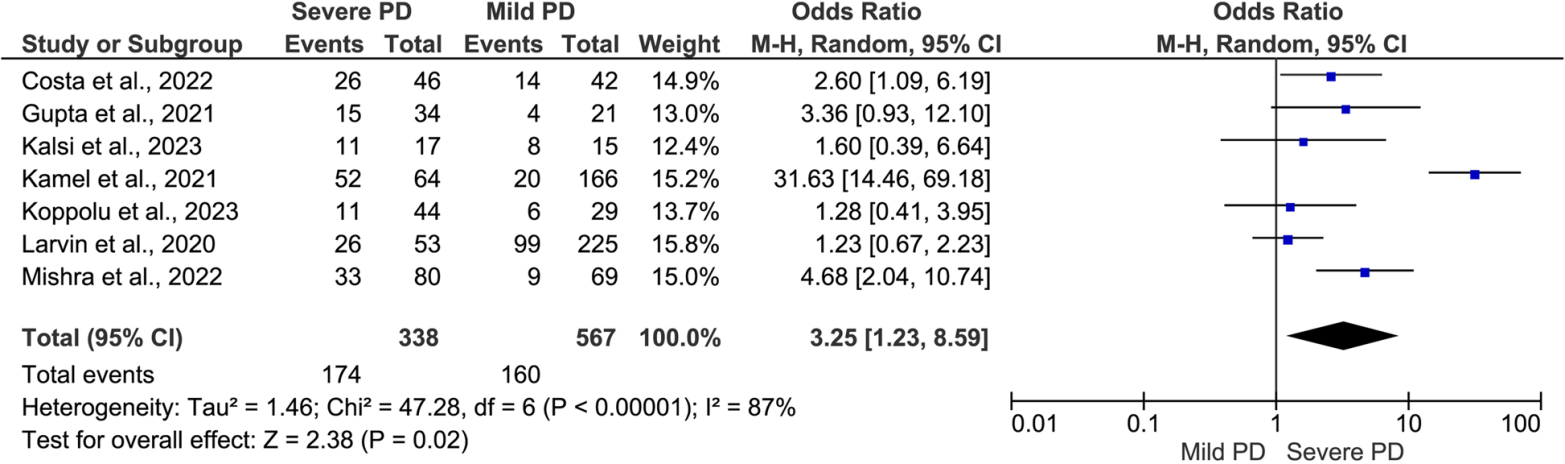
Meta-analysis of the association between severe periodontal disease (PD) and COVID-19 symptoms

**Fig. 6:**
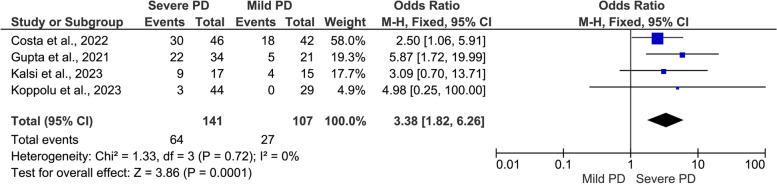
Meta-analysis of the association between severe periodontal disease (PD) and ICU admission

**Fig. 7:**
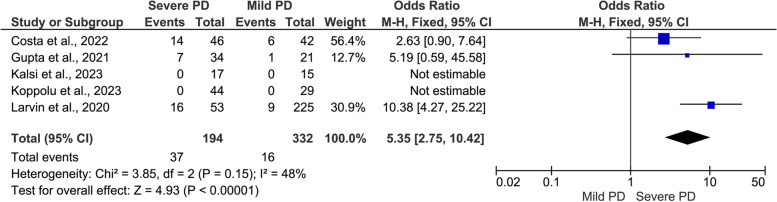
Meta-analysis of the association between severe periodontal disease (PD) and mortality rate

The results revealed a positive significant association between the severity of PD and severity of COVID-19 outcomes (Figs. 5, 6 and 7): severe symptoms (OR = 3.25, 95% CI: 1.23, 8.59, I^2^ = 87%, random-effect; *P* = 0.02) (Fig. 5); ICU admission (OR = 3.38, 95% CI: 1.82, 6.26, I^2^ = 0.00%, fixed-effect; *P* = 0.0001) (Fig. 6), and mortality rate (OR = 5.35, 95% CI: 2.75, 10.42, I^2^ = 48%, fixed-effect; *P* = 0.00001) (Fig. 7).

#### Risk of COVID-19 in PD patients versus healthy periodontium patients:

**Fig. 8:**
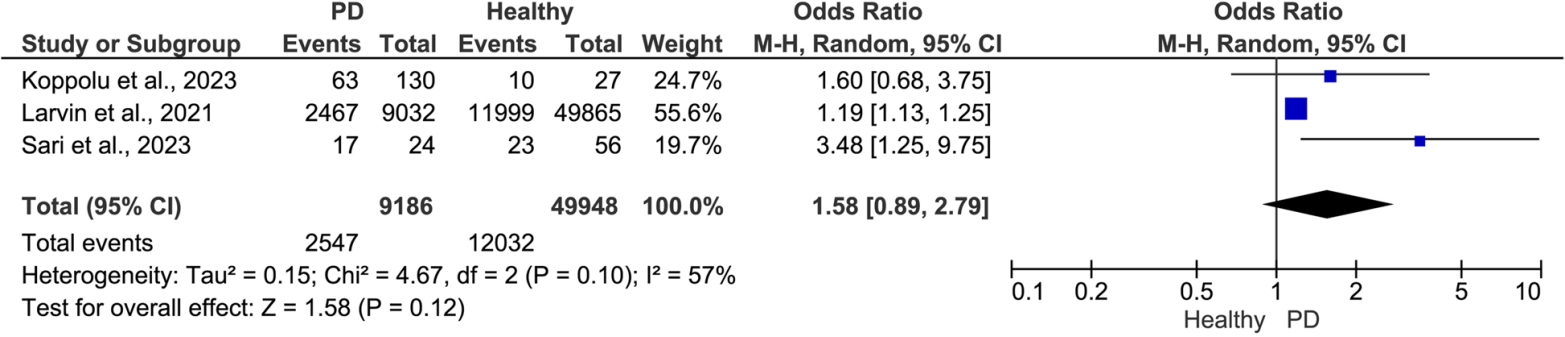
Meta-analysis of COVID-19 risk in relation to periodontal health status (PD vs. healthy)

The pooled three studies revealed a higher risk of COVID-19 infection in periodontitis patients (OR = 1.58, 95% CI: 0.89, 2.79, I^2^ = 57%, *P* = 0.12), although the result was not statistically significant (Fig. 8).

#### Publication bias

The funnel plots for publication bias of the include studies for all categories are presented in the Supplementary file 2. Generally, there was not a noticeable publication bias among the studies, except Fig. 1 in the Supplementary file 2 which showed slight publication bias for the association between periodontal disease (PD) and severe COVID-19 symptoms.

#### Quality assessment

The results of the quality assessment- based on NOS- are presented in Table 3. Overall, the included studies revealed relatively good quality ranging from five to nine stars. Fourteen studies [22–25, 27–29, 34, 36–38, 40, 43–45] were of high quality, while seven studies were of moderate quality [33, 35, 39, 41, 42, 46, 47]. The most frequent methodological shortcomings were related to the self-report ascertainment of the exposure and bias in selection of cases/controls (Table 3).

**Table 3:** NOS-based quality analysis of the included studies

**Study**	**Selection**	**Comparability**	**Exposure/Outcome**	**Overall**
Anand et al., 2021[22]	****	**	***	Low
Marouf et al., 2021 [25]	****	**	***	Low
Larvin et al 2020 [29]	***	**	***	Low
Gupta et al. 2021 [23]	***	**	**	Low
Holt et al. 2021 [40]	**	*	***	Moderate
Donders et al 2022 [31]	***	**	***	Low
Larvin et al 2021 [30]	***	**	**	Low
Costa 2022 [37]	***	**	**	Low
Kaur et al 2022 [42]	**	*	***	Moderate
Mishra et al 2022 [44]	***	**	**	Low
Kamel et al. 2021 [24]	***	*	*	Moderate
Sirin & Ozcelik 2021 [47]	**	*	**	Moderate
Koppolu et al., 2023 [43]	**	**	***	Low
Sari et al., 2023 [46]	**	*	**	Moderate
Said et al., 2023 [27]	****	**	***	Low
Alnomay et al., 2023 [35]	**	**	**	Moderate
Guardado-Luevanos et al., 2022 [38]	**	**	***	Low
Gujar et al., 2022 [39]	**	**	***	Low
Wadhwa et al. 2022 [26]	***	**	***	Low
Poyato-Borrego et al., 2023 [45]	****	**	***	Low
Kalsi et al., 2023 [41]	***	**	***	Low
Bemquerer et al., 2023 [36]	**	**	**	Moderate

## Discussion

Medical literature has linked PD to the risk and severity of COVID-19. Hence, the present systematic review and meta-analysis sought to answer the following focused question: Does PD influence the risk and severity of COVID-19? Qualitatively, most of the included studies reported significant association between PD and COVID-19 severity. However, three studies [27–29] failed to replicate these results. Quantitatively the pooled data found a significant positive association between PD and the risk and adverse outcomes of COVID-19 such as severe symptoms, ICU admission. Additionally, severe PD was significantly associated with higher risk of severe COVID-19 symptoms (*P* = 0.02), ICU admission (*P* = 0.0001), and mortality rate (*P* = 0.00001) compared to mild PD. Indeed, the results revealed that patients with PD have significantly 54% higher risk to getting COVID-19 infection. However, these findings should be interpreted with caution owing to the heterogeneity among the included studies as well as some methodological limitations, discussed in the following sections.

The findings of the present systematic review support the results of previous systematic reviews [30, 31]. Nevertheless, although the results of the current study are interesting, the mechanism(s) by which periodontitis aggravate(s) COVID-19 adverse outcomes still unclear so far. However, many theories have been suggested, and deserve discussion. With regard to one of the key findings of the current study: association of PD and COVID-19 adverse outcomes, one possible explanation is related to the expression of angiotensin converting enzyme 2 (the well- known receptor for SARS-CoV-2) by the inspired periopathogenes. This subsequently leads to production of inflammatory cytokines such as IL-6 and IL-8 in the lower respiratory tract, thus aggravating the response [48]. Further, periopathogenes have been reported to enhance the virulence of SARS-CoV-2 by cleaving its S glycoproteins, a matter that exacerbates COVID-19 complications [49]. Of utmost important, the chronic inflammatory nature of periodontitis may play a role through triggering systemic inflammation, which aggravates the inflammatory response in context of many disease processes, and COVID-19 wouldn’t be an exception. A recent study reported existence of periopathogenes in the metagenome of patients severely infected with SARS-CoV-2: Mainly high reads for Prevotella (493 reads), Staphylococcus (1,659 reads) and Fusobacterium (463 reads) were discovered [50]. Indeed, the potential role of PD in pulmonary infections (and diseases) has long been investigated and well documented in the literature [16, 51–53]. Mounting evidence from systematic reviews and meta-analyses found a significant association between PD and exacerbation of respiratory conditions, mainly pneumonia and COPD [14, 17].

The significant association between PD and the risk of COVID-19 is another key and pivotal finding of the current study: patients with PD were 54% at higher risk of COVID-19 acquisition than people with healthy periodontium. Essentially, the oral cavity, including gingival pocket epithelium, has been reported to be potentially high risk for SARS-CoV-2 infectious susceptibility, mediated by expression of angiotensin converting enzyme 2 [54, 55]. These results might be explained by the role of periopathogenic bacteria in initiating the expression of angiotensin converting enzyme, as already discussed above. A very recent evidence suggests that periodontal pockets and decayed teeth serve as reservoirs for SARS-CoV-2, making people more prone to COVID-19 [56]. In addition to being hiding areas, where the antibacterial action of the saliva and mouth rinses is not effective, the periodontally involved pockets have higher surface area than that of normal gingival sulcus, providing more opportunities for SARS-CoV-2 to bind and eventually infect more enzyme-expressing cells, in addition to acting as reservoirs for continuing infection, or recurring, or complicating the current infection leading to a more severe disease. However, these are still hypotheses and more research are required to elucidate these phenomena.

Worthy to note that the positive association of PD with the outcomes of COVID-19 was supported by all of the included studies except two studies by Larvin et al., which failed to do so [27, 28]. It must be acknowledged that these two studies involved relatively large sample sizes of COVID-19 patients. However, the exposure (periodontal status) was self-reported, a matter which raises a lot of doubts and reduces reliability of these two studies. Obviously, it is a clear shortcoming, and therefore the results might have not accurately reflected the periodontal status of the participants, and thus might have biased the results. Apart from the fact that the number of present and/or the missing teeth can be self-reported to a large extent of reliability, the other periodontal health outcomes and parameters cannot be reported by the patients precisely [57] . This, in turn, may explain the different results obtained by these two studies, a matter that must be acknowledged too. By contrast, except for Kamel et al. study, all other studies, which reported positive association of PD with the outcomes of COVID-19 ascertained the periodontal parameters objectively, either clinically or using radiographs.

Undoubtedly, the quality of the individual studies is a very determining factor that influences the quality of the overall evidence of any meta-analysis [58]. For this purpose, two reviewers evaluated independently the quality of all included studies using NOS, a very valid risk assessment tool. The results revealed relatively good quality of the included studies. Selection bias and self-reporting of the exposure (periodontal parameters) were the most frequently shortcomings, which cause biased results: recapitulating and reminding Larvin et al. studies mentioned above.

The present systematic review has many points that add to its strength and should be recognized. First, the study included a good number of studies with a relatively large sample size. Second, the studies were conducted in different geographical areas representing the world and thus substantiating its external validity. However, there are few limitations that should be highlighted. The main limitation is the heterogeneity among the included studies in many respects including: study design, the assessed periodontal parameters (exposure), COVID-19 parameters (outcome), setting of the included patients (hospitalized vs non-hospitalized patients), age of the participants, and many other confounders. The differences in methods of measurements of periodontal parameters represent a marked heterogeneity, being self-reported in a few studies, using radiographs in some studies, and clinical examination in the other studies. In particular, the self-reporting of periodontal status is an evident drawback of the present study that might have caused bias in the results. Furthermore, missing of some of the numerical data was an obvious obstacle that hindered including all studies in the quantitative analysis.

## Conclusion

In conclusion, the present systematic review and meta-analysis suggests a significant association between poor periodontal health and poor COVID-19 outcomes. However, the results should be interpreted with caution given the marked heterogeneity across the included studies along with some methodological limitations in some of these studies. Hence, further large-scale prospective cohort studies with standardized methodologies are highly required to further elucidate the potential association between periodontal diseases and the risk of poor COVID-19 outcomes.

### Supplementary Information

**Additional file 1:** **Table S1.** Databases. Applied search strategy, and numbers of retrieved studies. **Table S2.** List of excluded studies and reason for exclusion. 

**Additional file 2:** **Figure S1.** Funnel plot for the included studies of the association between periodontal disease (PD) and severe COVID-19 symptoms. **Figure 2.** Funnel plot for the included studies of the association between periodontal disease (PD) and ICU admissions. **Figure S3.** Funnel plot for the included studies of the association between periodontal disease (PD) and mortality. **Figure S4.** Funnel plot for the included studies of the association between severe periodontal disease (PD) and COVID-19 symptoms. **Figure S5.** Funnel plot for the included studies of the association between severe periodontal disease (PD) and ICU admission. **Figure S6.** Funnel plot for the included studies of the association between severe periodontal disease (PD) and mortality rate. **Figure S7.** Funnel plot for the included studies of COVID-19 risk in relation to periodontal health status (PD vs. healthy).

## Data Availability

All data generated or analyzed during this study are included in this published article.

## Abbreviations

COVID-19: Coronavirus Disease 2019
PD: Periodontitis
ICU: Intensive Care Unit
CI: Confidence Interval
NOS: Newcastle Ottawa Scale
OR: Odds Ratio
