# Supplementary material for: The impact of periodontal disease on the clinical outcomes of COVID-19: A systematic review and meta-analysis
Source: BMC Oral Health. 2023 Sep 9;23:658. doi: 10.1186/s12903-023-03378-0 (PMC10493030; doi:10.1186/s12903-023-03378-0)
Supplement: Supplementary file 1 — Additional file 1: Table S1. Databases. Applied search strategy, and numbers of retrieved studies. Table S2. List of excluded studies and reason for exclusion. [file 12903_2023_3378_MOESM1_ESM.docx]

| Supplementary Table 1. Databases. Applied search strategy, and numbers of retrieved studies. | | |
| --- | --- | --- |
|  | | |
| **Database of published trials, dissertations and conference proceedings** | **Search strategy used** | **Hits** |
| MEDLINE searched via PubMed on June 1st, 2022, and updated on April 16^th^, 2023 via www.ncbi .nlm.nih.gov/sites | #1 search: ("Periodontal Diseases"[MeSH Terms] OR "Oral Health"[MeSH Terms] OR periodontitis OR "periodont*"[All Fields] OR "periodontal pathogens" [All Fields]) 29,161  #2 search: ("SARS-CoV-2"[MeSH Terms] OR ("covid 19"[All Fields] OR "covid 19"[MeSH Terms] OR "covid 19 vaccines"[All Fields] OR "covid 19 vaccines"[MeSH Terms] OR "covid 19 serotherapy"[All Fields] OR "covid 19 nucleic acid testing"[All Fields] OR "covid 19 nucleic acid testing"[MeSH Terms] OR "covid 19 serological testing"[All Fields] OR "covid 19 serological testing"[MeSH Terms] OR "covid 19 testing"[All Fields] OR "covid 19 testing"[MeSH Terms] OR "SARS-CoV-2"[All Fields] OR "SARS-CoV-2"[MeSH Terms] OR "severe acute respiratory syndrome coronavirus 2"[All Fields] OR "ncov"[All Fields] OR "2019 ncov"[All Fields] OR (("coronavirus"[MeSH Terms] OR "coronavirus"[All Fields] OR "cov"[All Fields]) AND 2019/11/01:3000/12/31[Date - Publication]))) AND (2019:2023[pdat]) 355,206  # 3 search: #1 AND #2 AND  878 | 878 |
| ISI web of science Core Collection was searched via web of knowledge on June 1st, 2022, and updated on April 16^th^, 2023 via apps.webofknowledge.com | # 1 (( "oral health" OR "Periodontal disease" OR periodontitis OR "periodontal pathogens") AND ("SARS-CoV-2" OR COVID-19))  (All Fields) 749 | 749 |
| Scopus searched on June 1st, 2022, and updated on April 16^th^, 2023 via https://www.scopus.com | **# 1 search** TITLE-ABS-KEY ( "Periodontal Diseases" OR "Periodontitis" OR "Oral Health" OR "gingivitis" ) AND PUBYEAR > 2018 AND PUBYEAR < 2024 **32,142**  # 2 search : TITLE-ABS-KEY ( "SARS-CoV-2" OR covid-19) AND PUBYEAR > 2018 AND PUBYEAR < 2024  461,534  Search # 3: #1 AND #2 875 | 875 |
| Google Scholar searched on June 5, 2022 and updated on April 16^th^, 2023 via <https://scholar.google.com/> | (("Periodontal disease" OR periodontitis OR "oral health") AND ("SARS-CoV-2" OR COVID-19)) 23, 500 | 500 |
| Total |  |  |
|  |  | 3002 |

Supplementary Table 2: List of excluded studies and reason for exclusion

| **#** | **Reference** | **Reason for exclusion** |
| --- | --- | --- |
|  | Tamimi F, Altigani S, Sanz M. Periodontitis and coronavirus disease 2019. Periodontol 2000. 2022 Jun;89(1):207-214. doi: 10.1111/prd.12434. Epub 2022 Mar 4. PMID: 35244975; PMCID: PMC9115349. | Review |
|  | Patel J, Sampson V. The role of oral bacteria in COVID-19. Lancet Microbe. 2020 Jul;1(3):e105. doi: 10.1016/S2666-5247(20)30057-4. Epub 2020 Jul 3. PMID: 32835339; PMCID: PMC7333982. | Correspondence |
|  | Shamsoddin E. Is periodontitis associated with the severity of COVID-19? Evid Based Dent. 2021 Jan;22(2):66-68. doi: 10.1038/s41432-021-0179-x. PMID: 34172910; PMCID: PMC8226334. | commentary |
|  | Adam M. Is SARS-CoV-2 present in the periodontium? A post-mortem study. Evid Based Dent. 2021 Jan;22(2):60-61. doi: 10.1038/s41432-021-0184-0. PMID: 34172907; PMCID: PMC8226333. | commentary |
|  | Kara C, Çelen K, Dede FÖ, Gökmenoğlu C, Kara NB. Is periodontal disease a risk factor for developing severe Covid-19 infection? The potential role of Galectin-3. Exp Biol Med (Maywood). 2020 Oct;245(16):1425-1427. doi: 10.1177/1535370220953771. Epub 2020 Aug 24. PMID: 32838557; PMCID: PMC7553094. | Commentary |
|  | Fernandes Matuck B, Dolhnikoff M, Maia GVA, Isaac Sendyk D, Zarpellon A, Costa Gomes S, Duarte-Neto AN, Rebello Pinho JR, Gomes-Gouvêa MS, Sousa SCOM, Mauad T, Saldiva PHDN, Braz-Silva PH, da Silva LFF. Periodontal tissues are targets for Sars-Cov-2: a post-mortem study. J Oral Microbiol. 2020 Nov 26;13(1):1848135. doi: 10.1080/20002297.2020.1848135. PMID: 33391625; PMCID: PMC7717160. | Post-mortem study |
|  | Takahashi Y, Watanabe N, Kamio N, Yokoe S, Suzuki R, Sato S, Iinuma T, Imai K. Expression of the SARS-CoV-2 Receptor ACE2 and Proinflammatory Cytokines Induced by the Periodontopathic Bacterium *Fusobacterium nucleatum* in Human Respiratory Epithelial Cells. Int J Mol Sci. 2021 Jan 29;22(3):1352. doi: 10.3390/ijms22031352. PMID: 33572938; PMCID: PMC7866373. | Experimental study |
|  | Cai, W., Marouf, N., Said, K.N. and Tamimi, F., 2021. Nature of the Interplay Between Periodontal Diseases and COVID-19. *Frontiers in Dental Medicine*, p.87 | Review |
|  | Madapusi Balaji T, Varadarajan S, Rao USV, Raj AT, Patil S, Arakeri G, Brennan PA. Oral cancer and periodontal disease increase the risk of COVID 19? A mechanism mediated through furin and cathepsin overexpression. Med Hypotheses. 2020 Nov;144:109936. doi: 10.1016/j.mehy.2020.109936. Epub 2020 Jun 1. PMID: 32505073; PMCID: PMC7263251. | Letter to Editors |
|  | Sahni V, Gupta S. COVID-19 & Periodontitis: The cytokine connection. Med Hypotheses. 2020 Nov;144:109908. doi: 10.1016/j.mehy.2020.109908. Epub 2020 May 30. PMID: 32534336; PMCID: PMC7832148. | Letter to Editors |
|  | Badran Z, Gaudin A, Struillou X, Amador G, Soueidan A. Periodontal pockets: A potential reservoir for SARS-CoV-2? Med Hypotheses. 2020 Oct;143:109907. doi: 10.1016/j.mehy.2020.109907. Epub 2020 May 30. PMID: 32504927; PMCID: PMC7833827. | Hypotheses |
|  | Elisetti N. Periodontal pocket and COVID-19: Could there be a possible link? Med Hypotheses. 2021 Jan;146:110355. doi: 10.1016/j.mehy.2020.110355. Epub 2020 Nov 1. PMID: 33183854; PMCID: PMC7604063. | Hypotheses |
|  | Utomo, H., Wijaksana, I.K.E. and Prahasanti, C., 2021. Porphyromonas gingivalis in Periodontitis: A forgotten enemy behind COVID-19 pandemic. *Dental Hypotheses*, *12*(1), p.28. | Hypotheses |
|  | Pitones-Rubio V, Chávez-Cortez EG, Hurtado-Camarena A, González-Rascón A, Serafín-Higuera N. Is periodontal disease a risk factor for severe COVID-19 illness? Med Hypotheses. 2020 Nov;144:109969. doi: 10.1016/j.mehy.2020.109969. Epub 2020 Jun 19. PMID: 32592918; PMCID: PMC7303044. | Hypotheses |
|  | Gupta S, Sahni V. The intriguing commonality of NETosis between COVID-19 & Periodontal disease. Med Hypotheses. 2020 Nov;144:109968. doi: 10.1016/j.mehy.2020.109968. Epub 2020 Jun 7. PMID: 32534340; PMCID: PMC7276117. | Hypotheses |
|  | Gupta S, Saarikko M, Pfützner A, Räisänen IT, Sorsa T. Compromised periodontal status could increase mortality for patients with COVID-19. Lancet Infect Dis. 2022 Mar;22(3):314. doi: 10.1016/S1473-3099(22)00065-2. PMID: 35218744; PMCID: PMC8865881. | Correspondence |
|  | Andrade, R.M., Marques, R.S., de Moura, T.R., de Paiva, S.M., Gurgel, R.Q. and Martins-Filho, P.R., 2021. Is there a bidirectional interaction between periodontitis and the severity of SARS-CoV-2 infection?. *EXCLI journal*, *20*, p.1009. | Letter to Editors |
|  | Natto ZS, Afeef M, Bakhrebah MA, Ashi H, Alzahrani KA, Alhetheel AF, Fletcher HM. Can periodontal pockets and caries lesions act as reservoirs for coronavirus? Mol Oral Microbiol. 2022 Apr;37(2):77-80. doi: 10.1111/omi.12362. Epub 2022 Feb 4. PMID: 35060684. | Irrelevant outcome |
|  | Morales A, Corral-Nuñez C, Galaz C, Henríquez L, Mery M, Mesa C, Strauss F, Cavalla F, Baeza M, Valenzuela-Villarroel F, Gamonal J. Impact of COVID-19 Pandemic on Quality of Life of Type II Diabetes Patients With Periodontitis. Front Oral Health. 2021 Jun 4;2:682219. doi: 10.3389/froh.2021.682219. PMID: 35048027; PMCID: PMC8757750. | irrelevant |
|  | Botros N, Iyer P, Ojcius DM. Is there an association between oral health and severity of COVID-19 complications? Biomed J. 2020 Aug;43(4):325-327. doi: 10.1016/j.bj.2020.05.016. Epub 2020 May 29. PMID: 32713780; PMCID: PMC7258848. | Hypotheses |
|  | Molayem, S. and Pontes, C.C., 2020. The Mouth-COVID Connection: H-6 Levels in Periodontal Disease—Potential Role in COVID-19-Related Respiratory Complications. *Medicina stomatologică*, *57*(4), pp.68-80. | Review |
|  | Gómez-Costa D, Ramírez JM, García Guerrero I, Giovannini G, Rojo R, Gómez-de Diego R. A retrospective study on the effect of the COVID-19 pandemic on dental treatments in adults. BMC Oral Health. 2022 Dec;22(1):1-8. | Irrelevant |
|  | Sampson V, Kamona N, Sampson A. Could there be a link between oral hygiene and the severity of SARS-CoV-2 infections? Br Dent J. 2020 Jun;228(12):971-975. doi: 10.1038/s41415-020-1747-8. PMID: 32591714; PMCID: PMC7319209. | Review |
|  | Beltrán V, Flores M, Sanzana C, Muñoz-Sepúlveda F, Alvarado E, Venegas B, Molina JC, Rueda-Velásquez S, von Marttens A. Tooth Loss and Caries Experience of Elderly Chileans in the Context of the COVID-19 Pandemic in Five Regions of Chile. Int J Environ Res Public Health. 2023 Feb 9;20(4):3001. doi: 10.3390/ijerph20043001. PMID: 36833696; PMCID: PMC9967189. | irrelevant |
|  | Takefuji Y. COVID and perio. Br Dent J. 2023 Feb;234(4):197. doi: 10.1038/s41415-023-5585-3. Epub 2023 Feb 24. PMID: 36828995; PMCID: PMC9951135. | Commentary |
|  | Favia G, Barile G, Tempesta A, Copelli C, Novielli G, Dell'Olio F, Capodiferro S, Spirito F, Brienza N, Ribezzi M, Vestito MC, Corriero A, Carpagnano E, Moschetta A, Chironna M, Loconsole D, Centrone F, Quadri MFA, Tartaglia GM, Limongelli L. Relationship between oral lesions and severe SARS-CoV-2 infection in intensive care unit patients. Oral Dis. 2023 Jan 20. doi: 10.1111/odi.14515. Epub ahead of print. PMID: 36680407. | Irrelevant |
|  | Bachtiar BM, Bachtiar EW, Kusumaningrum A, Sunarto H, Soeroso Y, Sulijaya B, Apriyanti E, Fragrantia Theodorea C, Putra Pratomo I, Yudhistira, Efendi D. *Porphyromonas gingivalis* association with inflammatory markers and exosomal miRNA-155 in saliva of periodontitis patients with and without diabetes diagnosed with COVID-19. Saudi Dent J. 2023 Jan;35(1):61-69. doi: 10.1016/j.sdentj.2022.12.002. Epub 2022 Dec 16. PMID: 36540394; PMCID: PMC9756571. | Irrelevant |
|  | AlAhmari F, Preethanath RS, Divakar DD, Ali D. Association of Peri-Implant Health Status With COVID-19. Int Dent J. 2022 Dec 1:S0020-6539(22)00275-1. doi: 10.1016/j.identj.2022.11.019. Epub ahead of print. PMID: 36642571; PMCID: PMC9712070. | Irrelevant outcome |
|  | Gardelis P, Zekeridou A, Suh N, Le Terrier C, Stavropoulos A, Giannopoulou C. A pilot clinical and radiographic study on the association between periodontitis and serious COVID-19 infection. Clin Exp Dent Res. 2022 Oct;8(5):1021-1027. doi: 10.1002/cre2.610. Epub 2022 Aug 6. PMID: 35932180; PMCID: PMC9562574. | Pilot study |
|  | Aly NM, Elwan AH, Elzayet RM, Hassanato NMR, Deif M, Abdelaziz WE, El Tantawi M. Association between COVID-19 stress, coping mechanisms and stress-related oral conditions among Egyptian adults: a cross-sectional study. Sci Rep. 2022 Oct 27;12(1):18062. doi: 10.1038/s41598-022-22961-z. PMID: 36302880; PMCID: PMC9610319. | irrelevant |
|  | Gupta S, Räisänen IT, Sorsa T. Periodontitis as a Risk of Hospitalization and Death by SARS-CoV-2. Int J Public Health. 2022 Aug 31;67:1605156. doi: 10.3389/ijph.2022.1605156. PMID: 36119447; PMCID: PMC9472290. | Commentary |
|  | Abbas H, Takeuchi K, Koyama S, Osaka K, Tabuchi T. Association Between Toothbrushing Habits and COVID-19 Symptoms. Int Dent J. 2023 Apr;73(2):302-310. doi: 10.1016/j.identj.2022.07.011. Epub 2022 Aug 4. PMID: 36192224; PMCID: PMC9350673. | Irrelevant |
|  | Wang Y, Deng H, Pan Y, Jin L, Hu R, Lu Y, Deng W, Sun W, Chen C, Shen X, Huang XF. Periodontal disease increases the host susceptibility to COVID-19 and its severity: a Mendelian randomization study. J Transl Med. 2021 Dec 24;19(1):528. doi: 10.1186/s12967-021-03198-2. PMID: 34952598; PMCID: PMC8708510. | mendelian randomization |
|  | Meng Z, Ma Y, Li W, Deng X. Association between periodontitis and COVID-19 infection: a two-sample Mendelian randomization study. PeerJ. 2023 Jan 25;11:e14595. doi: 10.7717/peerj.14595. PMID: 36718446; PMCID: PMC9884046. | mendelian randomization |
|  | Zhang C, Sun Y, Xu M, Shu C, Yue Z, Hou J, Ou D. Potential links between COVID-19 and periodontitis: a bioinformatic analysis based on GEO datasets. BMC Oral Health. 2022 Nov 21;22(1):520. doi: 10.1186/s12903-022-02435-4. PMID: 36414950; PMCID: PMC9682728. | Genetic study |
