# Supplementary material for: The impact of periodontal disease on the clinical outcomes of COVID-19: A systematic review and meta-analysis
Source: BMC Oral Health. 2023 Sep 9;23:658. doi: 10.1186/s12903-023-03378-0 (PMC10493030; doi:10.1186/s12903-023-03378-0)
Supplement: Supplementary file 2 — Additional file 2: Figure S1. Funnel plot for the included studies of the association between periodontal disease (PD) and severe COVID-19 symptoms. Figure 2. Funnel plot for the included studies of the association between periodontal disease (PD) and ICU admissions. Figure S3. Funnel plot for the included studies of the association between periodontal disease (PD) and mortality. Figure S4. Funnel plot for the included studies of the association between severe periodontal disease (PD) and COVID-19 symptoms. Figure S5. Funnel plot for the included studies of the association between severe periodontal disease (PD) and ICU admission. Figure S6. Funnel plot for the included studies of the association between severe periodontal disease (PD) and mortality rate. Figure S7. Funnel plot for the included studies of COVID-19 risk in relation to periodontal health status (PD vs. healthy). [file 12903_2023_3378_MOESM2_ESM.docx]

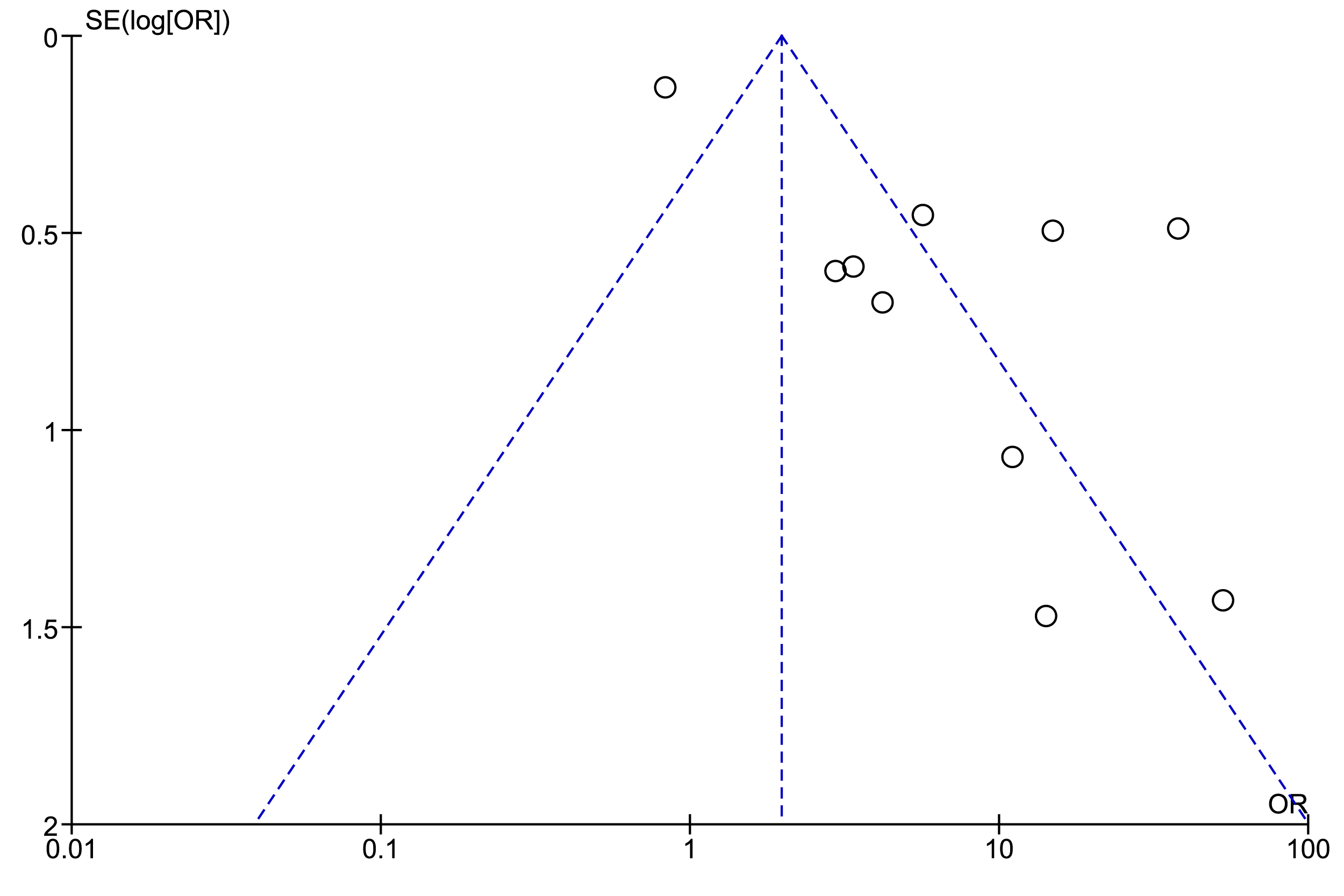


Supplementary Figure 1: Funnel plot for the included studies of the association between periodontal disease (PD) and severe COVID-19 symptoms


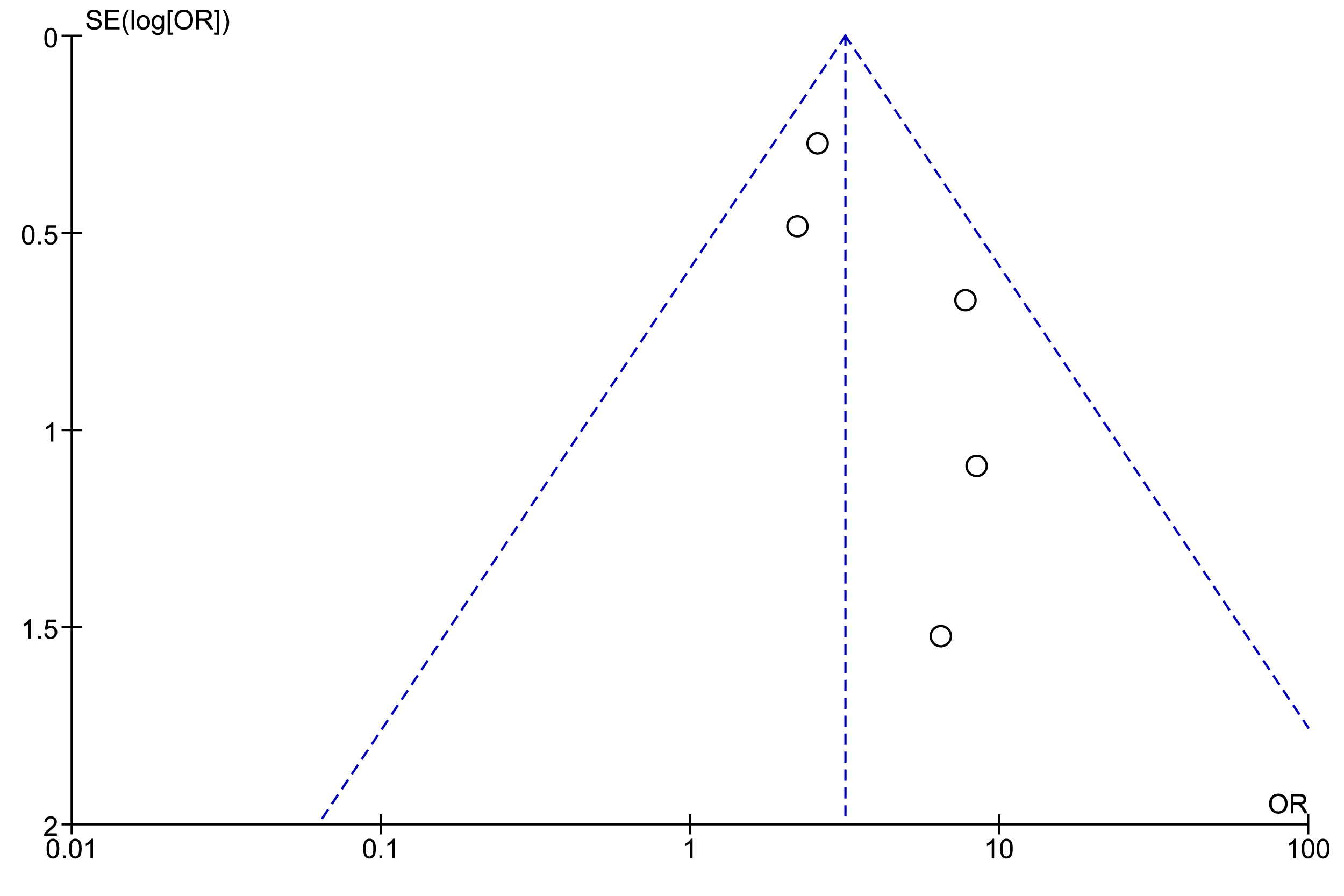


Supplementary Figure 2: Funnel plot for the included studies of the association between periodontal disease (PD) and ICU admissions


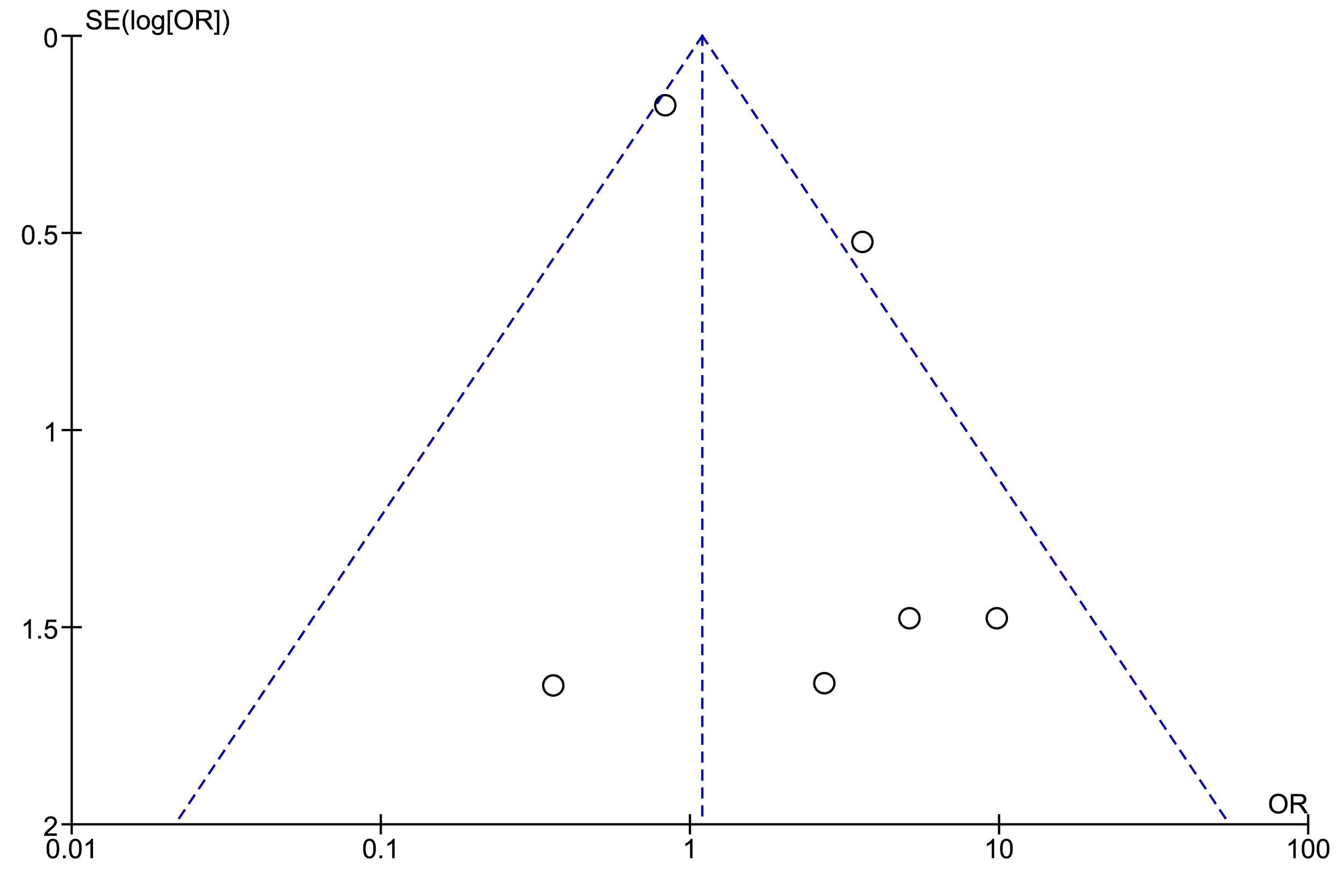


Supplementary Figure 3: Funnel plot for the included studies of the association between periodontal disease (PD) and mortality


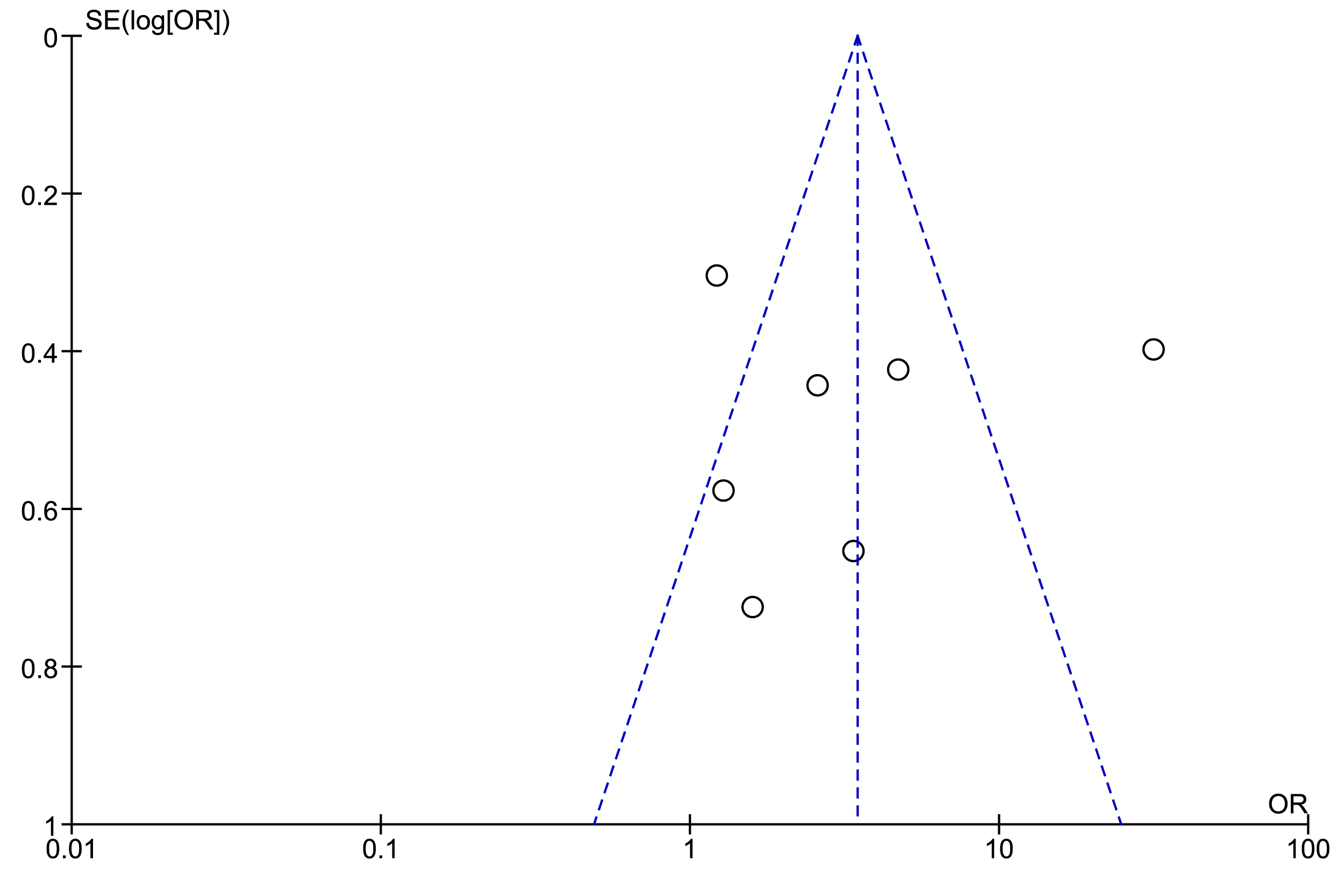


Supplementary Figure 4: Funnel plot for the included studies of the association between severe periodontal disease (PD) and COVID-19 symptoms


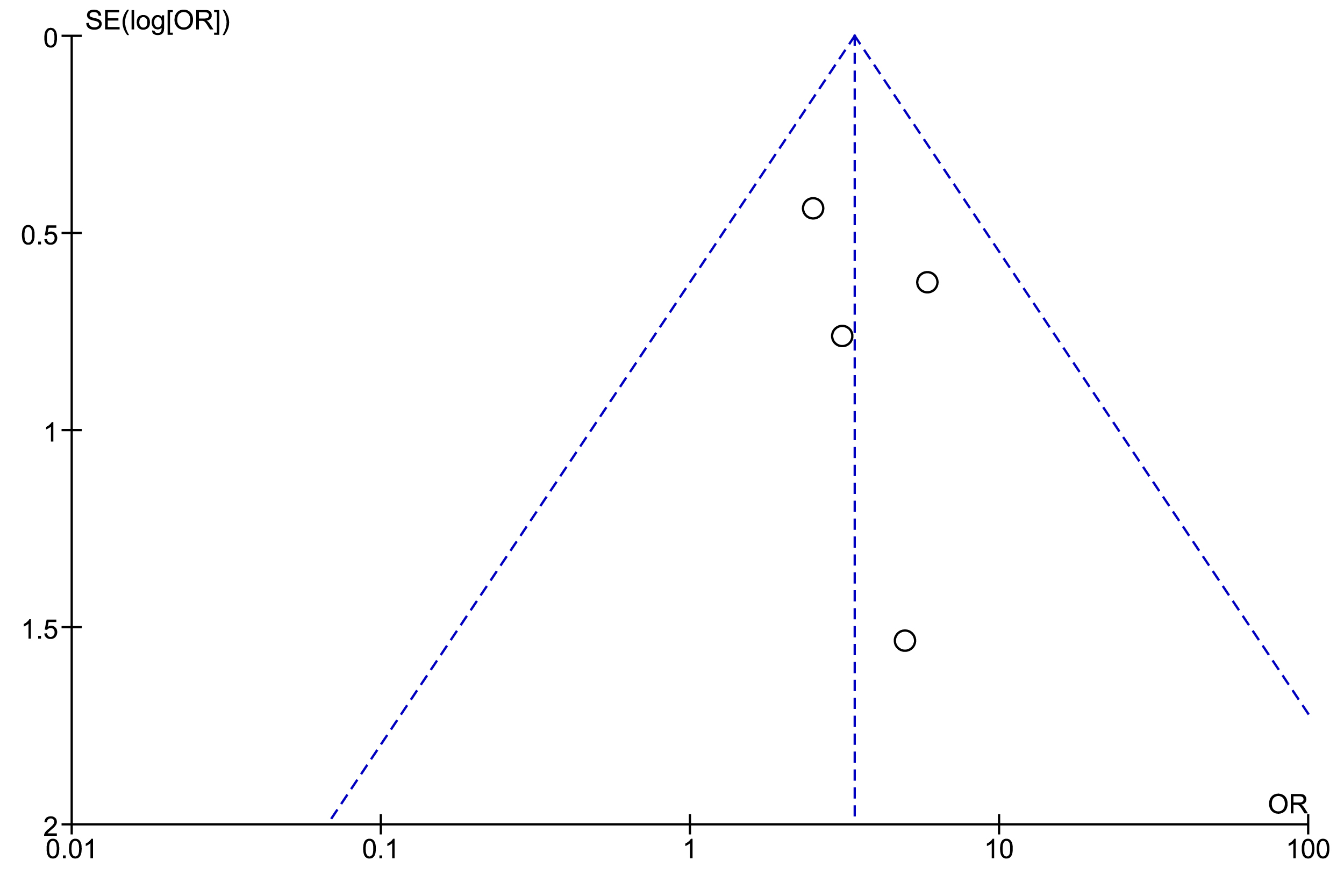


Supplementary Figure 5: Funnel plot for the included studies of the association between severe periodontal disease (PD) and ICU admission


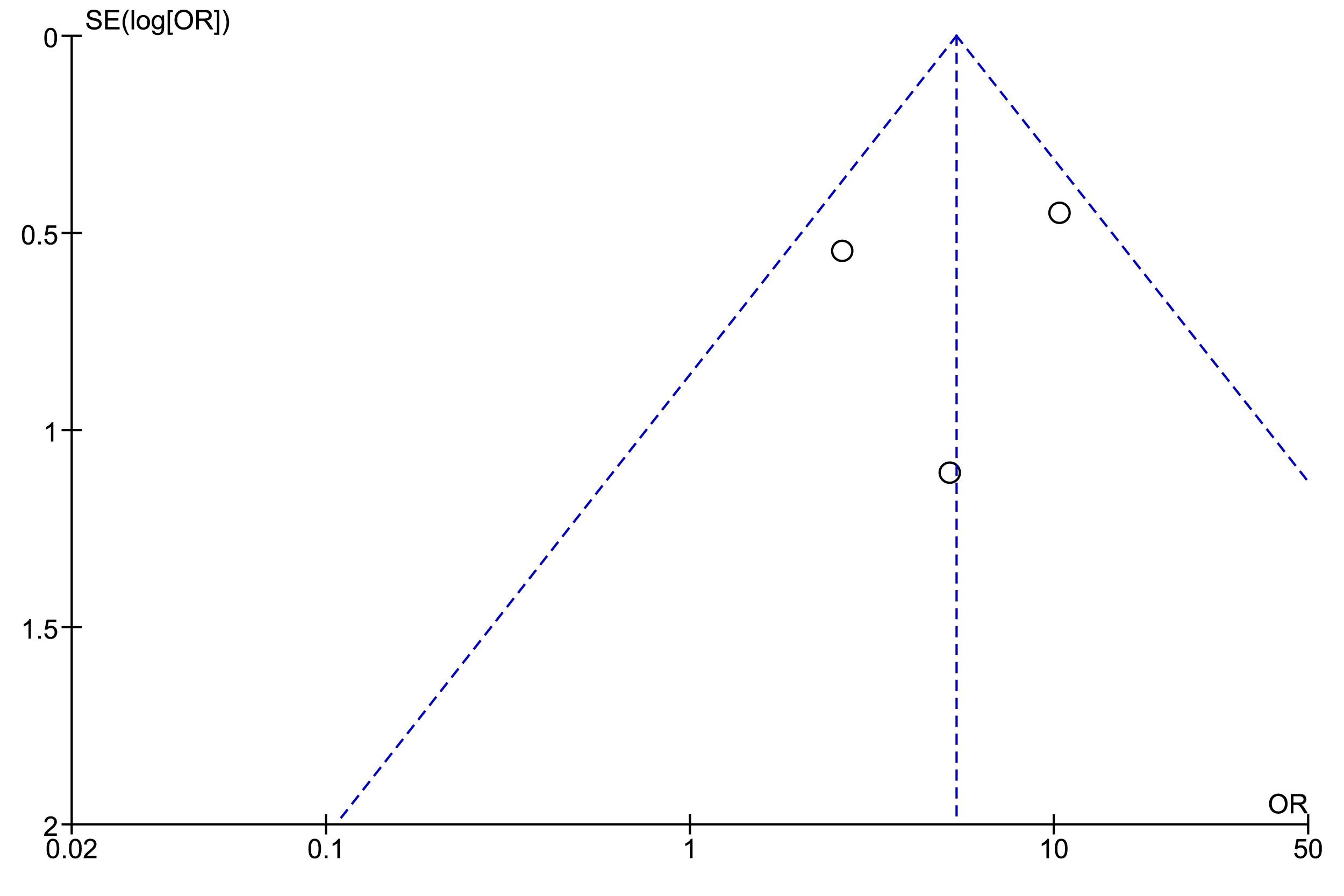


Supplementary Figure 6: Funnel plot for the included studies of the association between severe periodontal disease (PD) and mortality rate


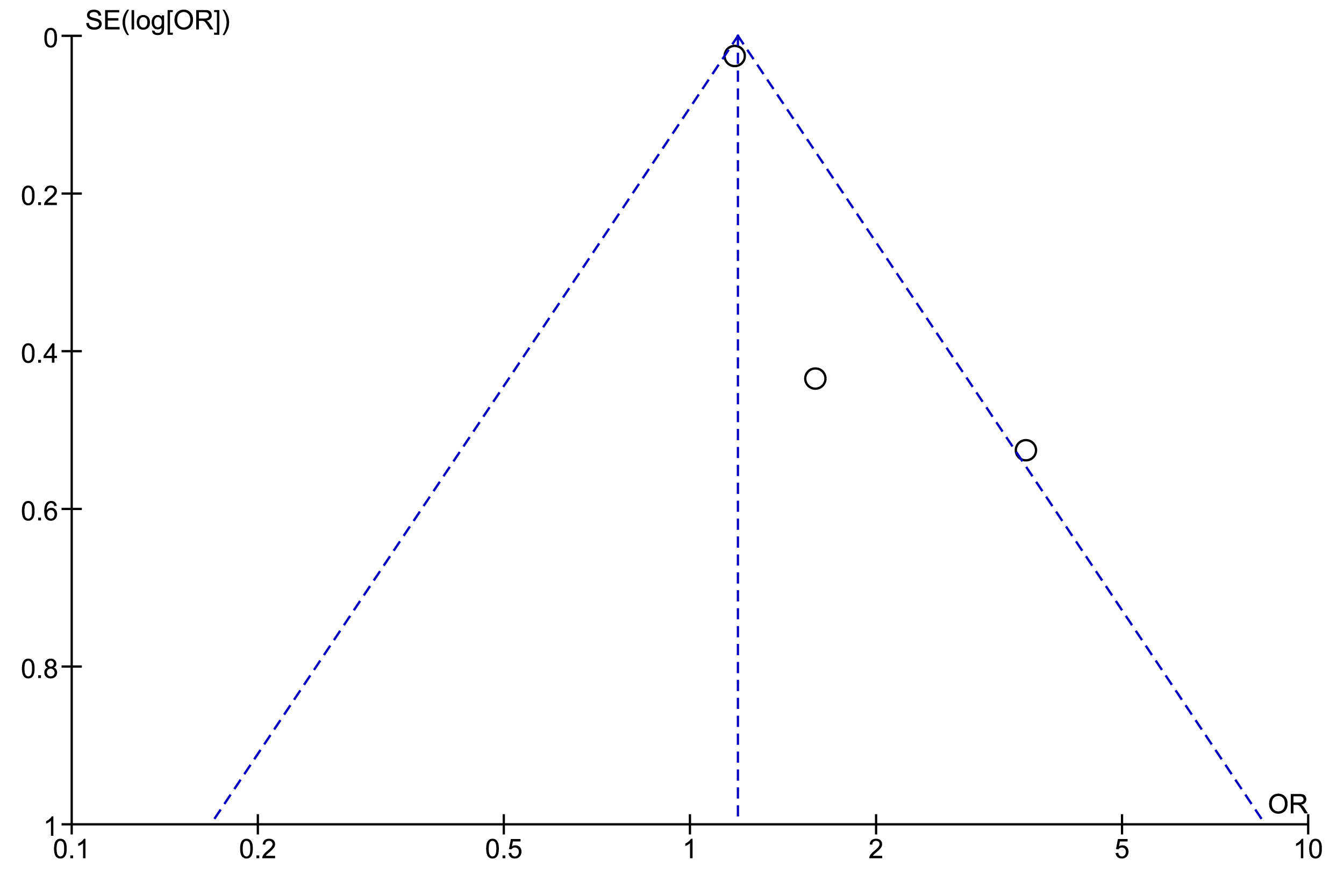


Supplementary Figure 7: Funnel plot for the included studies of COVID-19 risk in relation to periodontal health status (PD vs. healthy)
